# The complete mitochondrial genome of the jewel beetle *Coraebus cavifrons* (Coleoptera: Buprestidae)

**DOI:** 10.1080/23802359.2019.1636730

**Published:** 2019-07-12

**Authors:** Liangming Cao, Xiaoyi Wang

**Affiliations:** The Key Laboratory of Forest Protection, State Forestry and Grassland Administration of China, Research Institute of Forest Ecology, Environment and Protection, Chinese Academy of Forestry, Beijing, China

**Keywords:** Mitochondrial genome, Elateriformia, Buprestidae, *Coraebus cavifrons*

## Abstract

The complete mitochondrial genome (mitogenome) of the jewel beetle, *Coraebus cavifrons*, was sequenced and described in the present study. The mitogenome of *Coraebus cavifrons* is a typical circular DNA molecule of 15,686 bp. All of the 37 mitochondrial genes (13 protein-coding genes, 2 rRNA genes, and 22 tRNA genes) were annotated and a putative control region with 1135 bp in length was found between *tRNA^Ile^* and *srRNA*. Protein-coding genes all initiate with ATN codons except for *COII* uses GTG. Most of the protein-coding genes use TAA or TAG as the stop codon, but *COII*, *COIII,* and *ND5* terminate with a single T. All tRNAs have the clover-leaf structure except for *tRNA^Ser(AGN)^* and the length of them range from 60 to 71 bp. Genome organization and nucleotide composition of the mitogenome were also noted. Our phylogenetic analysis of Elateriformia supported the monophyly of Buprestoidea and the sister relationship between Buprestoidea and (Byrrhoidea + Elateroidea).

The genus *Coraebus* Gory & Laporte de Castelnau (Coleoptera: Buprestidae: Agrilinae: Coraebini: Coraebina) is a diversified group with a wide distribution (Xu et al. 2013). Two hundred twenty eight species were registered around the world, 108 were recorded in China (Kubáň 2006; Bellamy 2008). Among them, *Coraebus cavifrons* was firstly described by Descarpentries and Villiers (1967) and can be collected from South China. Herein, we sequenced the complete mitogenome of this beetle, *Coraebus cavifrons*, which is the first representation of *Coraebus*. Voucher specimen (No. VCim-00101) was deposited at the Entomological Museum of Chinese Academy of Forestry and the sequence was submitted to GenBank under the accession number MK913589.

The complete mitochondrial genome of *Coraebus cavifrons* is a circular DNA with 15,686 bp in length and includes 37 genes (13 protein-coding genes, 22 tRNA genes, and 2 rRNA genes) and a long non-coding region called control region. Gene organization is the same as the putative ancestral gene order without gene rearrangement (Clary and Wolstenholme 1985; Cameron 2014). Except for the control region, seven intergenic regions were found in this mitogenome ranging from 1 bp to 22 bp. There are a total of 78 bp overlapped nucleotides between adjacent genes in 19 locations, the longest is 17 bp between *ND4* and *tRNA^His^.*

The nucleotide composition of this mitogenome is significantly AT-biased. The A + T content is 69.8% with positive AT-skew (0.12) and negative CG-skew (-0.18). Protein-coding genes all initiate with ATN codons except for *COII* uses GTG. Most of the protein-coding genes use TAA (*ATP6*, *COI*, *ND1*, *ND2*, *ND4*, *ND4L*, *ND6*) or TAG (*ATP8*, *CYTB*, *ND3*) as the stop codon, but *COII*, *COIII,* and *ND5* terminate with a single T. Using a single T, stop codon is commonly found in many insect mitogenomes (Hong et al. 2009; Chen et al. 2018; Linghu et al. 2018).

This mitogenome contains all set of typical 22 tRNA genes present in animal mitochondrial genomes, ranging from 60 to 71 bp in length. Due to the deficiency of the dihydrouridine (DHU) arm, *tRNA^Ser(AGN)^* cannot be folded into the clover-leaf secondary structure like other 21 tRNAs. The *tRNA^Ser(AGN)^* with a simply looped DHU arm is also a common case in most insects (Li et al. 2012; Song et al. 2016). The *lrRNA* is 1265 bp long with an A + T content of 77.1% and the *srRNA* is 803 bp long with an A + T content of 71.7%. The control region is located between *tRNA^Ile^* and *srRNA* with 1135 bp in length. It shows significantly AT bias (75.8%) and contains a 14 bp poly-T.

Phylogenetic tree of Elateriformia based on the dataset of 13 PCGs using maximum likelihood method is shown in Figure 1. The result confirmed the monophyly of the four superfamilies within Elateriformia. Scirtoidea is tended to place at the basal position of the tree and Buprestoidea is the sister group to (Byrrhoidea + Elateroidea). These results are largely congruent with recently published hypotheses on the phylogenetic relationships among Elateriformia based on molecular data (Bocakova et al. 2007; Kundrata et al. 2017).

**Figure 1.**
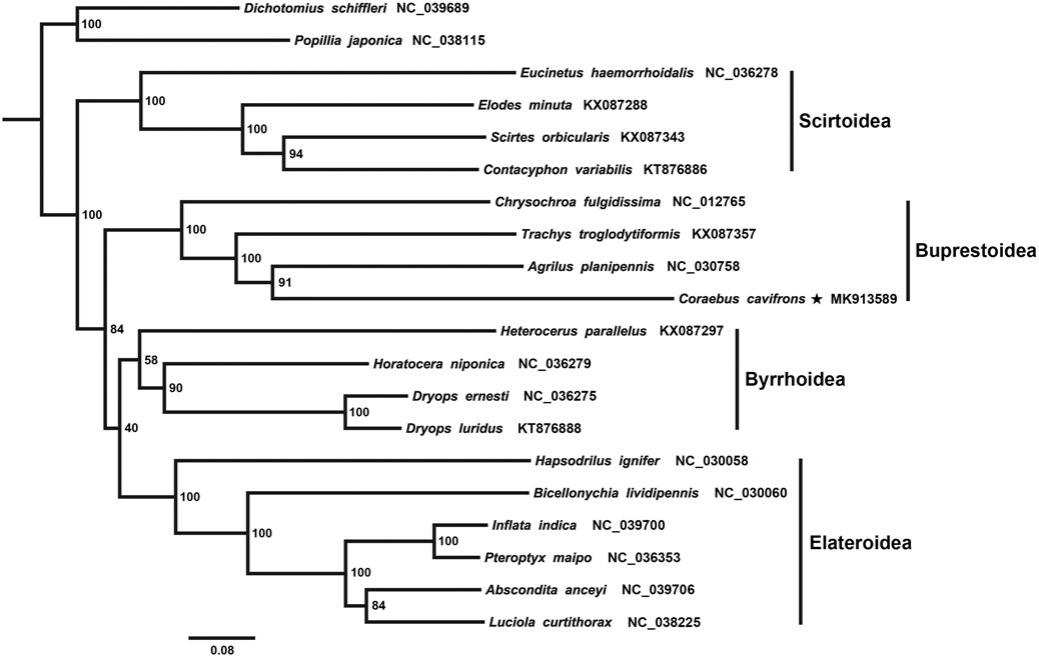
Phylogenetic relationship of 18 species among Elateriformia. Phylogenetic tree was conducted by ML analysis of the 13 protein-coding genes (10,890 bp) with IQ-TREE 1.6.5 (Trifinopoulos et al. 2016). The nodal values indicate the bootstrap percentages obtained with 1000 replicates. Alphanumeric terms indicate the GenBank accession numbers.
